# Artificial Intelligence for Adenoma and Polyp Detection During Screening and Surveillance Colonoscopy: A Randomized-Controlled Trial

**DOI:** 10.3390/jcm14020581

**Published:** 2025-01-17

**Authors:** Ali A. Alali, Ahmad Alhashmi, Nawal Alotaibi, Nargess Ali, Maryam Alali, Ahmad Alfadhli

**Affiliations:** 1 Department of Medicine, Faculty of Medicine, Kuwait University, Jabriyah 13110, Kuwait; 2 Thunayan Alghanim Gastroenterology Center, Amiri Hospital, Sharq 15300, Kuwait; 3 Department of Medicine, Jaber Alahmad Hospital, Zahra 47761, Kuwait; 4 Haya Al-Habeeb Gastroenterology Center, Mubarak Alkabeer Hospital, Jabriyah 13110, Kuwait

**Keywords:** colon cancer, CADe, colonoscopy, adenoma, polyp

## Abstract

**Background:** Colorectal cancer (CRC) is the second leading cause of cancer death in Kuwait. The effectiveness of colonoscopy in preventing CRC is dependent on a high adenoma detection rate (ADR). Computer-aided detection can identify (CADe) and characterize polyps in real time and differentiate benign from neoplastic polyps, but its role remains unclear in screening colonoscopy. **Methods:** This was a randomized-controlled trial (RCT) enrolling patients 45 years of age or older presenting for outpatient screening or surveillance colonoscopy (Kuwait clinical trial registration number 2047/2022). Patients with a history of inflammatory bowel disease, alarm symptoms, familial polyposis syndrome, colon resection, or poor bowel preparation were excluded. Patients were randomly assigned to either high-definition white-light (HD-WL) colonoscopy (standard of care) or HD-WL colonoscopy with the CADe system. The primary outcome was ADR. The secondary outcomes included polyp detection rate (PDR), adenoma per colonoscopy (APC), polyp per colonoscopy (PPC), and accuracy of polyp characterization. **Results:** From 1 September 2022 to 1 March 2023, 102 patients were included and allocated to either the HD-WL colonoscopy group (n = 51) or CADe group (n = 51). The mean age was 52.8 years (SD 8.2), and males represented 50% of the cohort. Screening for CRC accounted for 94.1% of all examinations, while the remaining patients underwent surveillance colonoscopy. A total of 121 polyps were detected with an average size of 4.18 mm (SD 5.1), the majority being tubular adenomas with low-grade dysplasia (47.1%) and hyperplastic polyps (46.3%). There was no difference in the overall bowel preparation, insertion and withdrawal times, and adverse events between the two arms. ADR (primary outcome) was non-significantly higher in the CADe group compared to the HD colonoscopy group (47.1% vs. 37.3%, *p* = 0.3). Among the secondary outcomes, PDR (78.4% vs. 56.8%, *p* = 0.02) and PPC (1.35 vs. 0.96, *p* = 0.04) were significantly higher in the CADe group, but APC was not (0.75 vs. 0.51, *p* = 0.09). Accuracy in characterizing polyp histology was similar in both groups. **Conclusions:** In this RCT, the artificial intelligence system showed a non-significant trend towards improving ADR among Kuwaiti patients undergoing screening or surveillance colonoscopy compared to HD-WL colonoscopy alone, while it significantly improved the detection of diminutive polyps. A larger multicenter study is required to detect the true effect of CADe on the detection of adenomas.

## 1. Introduction

Colorectal cancer (CRC) represents a significant public health problem, being the third most common malignancy overall and the second most fatal cancer globally [1,2,3]. In Kuwait, CRC is the second most common malignancy in males and females, and it accounts for more than 10% of all cancer-related mortality [4]. Most CRC cases develop from small adenomatous polyps that undergo a series of genetic alterations ultimately leading to cancer, a process known as the adenoma–carcinoma sequence [5,6]. Early detection of precancerous lesions via screening has been shown to reduce the incidence and CRC-related mortality [7].

Colonoscopy is a well-established modality that allows the detection and removal of early precancerous lesions and is currently one of the recommended modalities for CRC screening [1]. By removing precancerous lesions, colonoscopy helps to prevent the progression of adenomas to invasive adenocarcinomas, ultimately leading to a reduced incidence of CRC and its associated mortality. However, this beneficial effect of colonoscopy depends on detection of precancerous lesions, and the absence of such lesions has been associated with an increased risk of post-colonoscopy CRC [2]. Similarly, a high adenoma detection rate (ADR) has been associated with a lower risk of CRC, and it is estimated that for every 1% increase in the ADR, the risk of developing CRC is reduced by 3% [3,4]. Other important metrics that may be associated with a reduced incidence of CRC include a high rate of adenoma per colonoscopy (APC), which is considered one of the important quality indicators for screening colonoscopy [5].

Recently, several artificial intelligence (AI)-based tools have been developed to guide endoscopists in detecting (computer-aided detection (CADe)) and characterizing (CADx) colonic polyps during colonoscopy. The impact of these AI tools on colonoscopy outcomes has been inconsistent across different studies, with some studies concluding that AI is associated with improved ADR [6,7], while others did not find an improvement in ADR with such systems [8,9]. Several meta-analyses that incorporated some of these studies concluded that AI probably has a beneficial effect on colonoscopy quality metrics by increasing the ADR by approximately 20% [10,11,12]. However, all included studies were conducted in Western or Asian countries, limiting the generalizability of such findings to other regions, especially the Middle East. To further understand the role of AI on CRC screening in Kuwait, we performed a randomized controlled trial (RCT) with the aim to measure the impact of such technology on ADR among the average-risk population undergoing screening or surveillance colonoscopy.

## 2. Methods

***Center and ethics:*** This study was a single-center, prospective, randomized clinical trial evaluating the use of CADe during colonoscopy. The study was conducted in Mubarak Alkabeer Hospital in Jaberyah, Kuwait, in the period between 1 September 2022 and 1 March 2023. All procedures were performed by experienced endoscopists who had performed at least 1000 colonoscopies. Ethical approval was obtained from the Ministry of Health to conduct this trial on 3 August 2022. The study was registered as a clinical trial in the Kuwaiti clinical trial registry (Registration number 2047/2022).

***Patient selection:*** Adults 45 years or older presenting for average-risk screening or surveillance colonoscopy were enrolled. Additional inclusion criteria included providing informed consent by the patient and adequate bowel preparation (defined as Boston Bowel Preparation (BBP) scoring system ≥ 2 in each examined segment (minimum overall score ≥ 6). Exclusion criteria included a history of inflammatory bowel disease (ulcerative colitis or Crohn’s disease), alarm symptoms (e.g., weight loss, rectal bleeding), known or suspected familial polyposis syndrome (such as Lynch syndrome or familial adenomatosis polyposis), a history of colon resection, and poor bowel preparation. Patients in whom the cecum was not reached were also excluded.

***Study design:*** Eligible patients were randomized (1:1) into two arms. Randomization was based on computer-generated randomization numbers. Allocation was concealed and kept in a sealed envelope, which was opened prior to starting the procedure. The study was single-blinded; the patient was blinded to randomization. The endoscopist was aware of the randomization arm as the endoscopic and CADe images were displayed on the same screen simultaneously. The “Consolidated Standards of Reporting Trails, CONSORT” guidelines were followed for the reporting of this study.

***Procedure:*** All patients received the same bowel preparation regimen (picosulphate-based split preparation). In the control arm, patients underwent standard high-definition white-light (HDWL) colonoscopy. In the intervention arm, all patients received HDWL colonoscopy examination with the assistance of a CADe system, which was switched on during the withdrawal phase. All procedures were performed using the ELUXEO 7000 endoscopy platform (Fujifilm Co., Tokyo, Japan).

Colonoscopy was performed under conscious sedation using a combination of midazolam and fentanyl. Cecal intubation and withdrawal times were measured using a stopwatch, pausing during therapeutic interventions (e.g., polypectomy). The polyp size was estimated using open biopsy forceps and defined as diminutive if its size was 5 mm or less. The polyp morphology was described according to the Paris classification. All polyps were removed using different endoscopic techniques including biopsy forceps (for polyps < 3 mm in size), cold-snare (for polyps 3–10 mm), and endoscopic mucosal resection (for polyps > 10 mm) and sent for histopathological examination.

***Artificial intelligence system:*** The CAD EYE system (Fujifilm Co.) was used in this study. The CAD EYE system provides real-time computer-assisted image analysis, which allows automatic polyp detection and characterization. In the intervention group, the CAD EYE system was activated by the click of a button on the endoscope. When the CAD EYE identifies a polyp, both a visual (blue box demarcating the suspected polyp) and an acoustic alarm pop up to notify the endoscopist. In addition, the CAD EYE is equipped with a polyp characterization function that provides a prediction of the histology of the visualized polyp as either “hyperplastic” or “neoplastic”.

***Histopathology:*** All visualized polyps were resected and sent for histopathological examination after fixation in a 10% buffered formalin solution. Each polyp was sent in a separate jar, with their location clearly labeled. All polyps were examined by expert pathologists in Mubarak Alkabeer hospital, who were blinded to the assigned examination mode. All lesions were classified according to the Vienna classification [13] and World Health Organization [14]. Advanced adenomas were defined as any adenoma that was at least 10 mm in size, had significant villous features, high-grade dysplasia, or early invasive cancer.

***Study outcomes:*** The primary outcome was “adenoma detection rate, ADR”, defined as the proportion of patients with at least one histologically proven adenomatous polyp. Secondary outcomes included “polyp detection rate, PDR”, defined as the proportion of patients in which at least one polyp was detected over the total number of colonoscopies, “adenoma per colonoscopy, APC” and “polyp per colonoscopy, PPC”, defined as the number of detected adenomas or polyps divided by the total number of screening colonoscopies, respectively. Other important measured outcomes included the accuracy of polyp characterization, adverse events, withdrawal time, and cecal intubation rate.

***Statistical analysis and sample size calculation:*** Descriptive statistics were carried out and reported as mean +/− standard deviation or percentage. Comparisons of proportions were performed using a two-sided chi-squared test or Fisher’s exact test, as appropriate. For all comparisons, a *p* value of ≤0.05 was considered statistically significant. Results are expressed using the risk ratio (RR) and 95% confidence interval (CI) for the primary outcome. An intention to treat analysis was performed for all comparisons. All analyses were performed using STATA software version 15.1 (STATA corp. LP, College Station, TX, USA).

To estimate the sample size, we used a previously reported ADR (10%) from an unselected Kuwaiti population [15]. We hypothesized that the CADe system would improve the ADR to a level similar to those reported in other countries (35%), which is a clinically important threshold [16]. To test this hypothesis with an alpha error of 0.05 and power of 80%, at least 86 patients (43 in each group) were required. Considering a 10% overall dropout rate, we planned to include at least 95 patients overall.

## 3. Results

**Figure 1:**
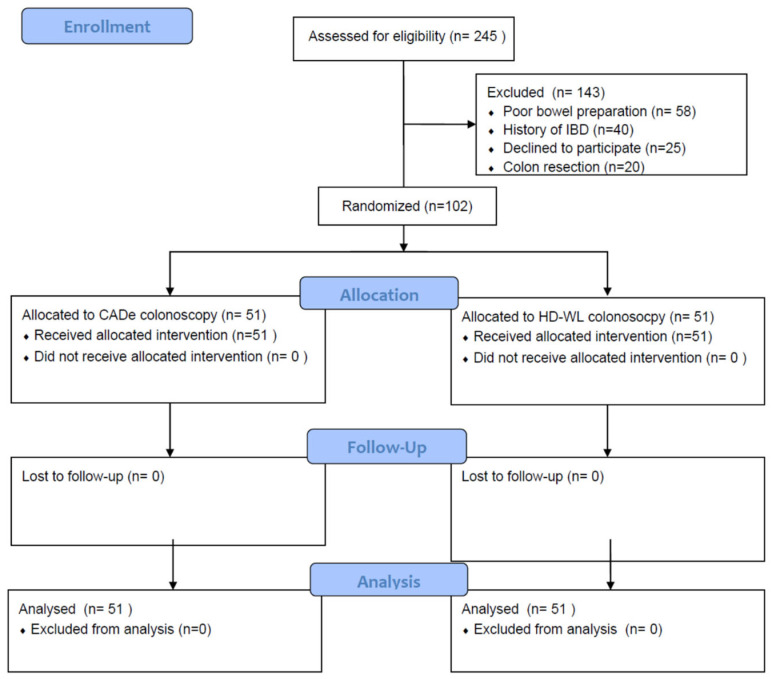
CONSORT flow diagram.

***Study population*:** A total of 245 patients were screened for inclusion, and 102 patients fulfilled the inclusion criteria and were enrolled in the study (51 patients in each arm) (CONSORT flow sheet, Figure 1). The mean age was similar between the CADe and the HD-WL colonoscopy groups (51.1 vs. 54.5 years, *p* = 0.9), but slightly more males were randomized to the CADe group (58.8% vs. 41.2%, *p* = 0.08). The distribution by indication was equal between the two groups (screening = 94.1%, surveillance = 5.9% in both groups). The bowel preparation scale was similar in both groups (BBPS = 8, *p* = 1.0), and a minority of patients were taking anithrombotics (CADe = 5.9%, HD-WL = 3.9%, *p* = 0.6). The insertion time was similar in the CADe and HD-WL groups (322.5 vs. 359.9 s, respectively, *p* = 0.8), as was the withdrawal time (542.4 vs. 509.4 s, respectively, *p* = 0.07). No adverse events were recorded (Table 1).

***Polyp characteristics:*** Overall, 121 polyps were detected and resected during the study period. The majority of detected polyps were small (mean size = 4.18 mm (SD 5.1)) with no difference between the two groups in terms of polyp size. The most common histology of resected polyps was tubular adenoma with low-grade dysplasia (47.1%), while hyperplastic polyps were the second most common type (46.3%). Other types of polyps detected are shown in Table 2. No malignant polyps or lesions were detected during the study period. There was no difference between the histological types of polyps detected between the two arms (*p* = 0.8). The majority (83.5%) of polyps were sessile with minimal elevation (Paris 0-IIa) and distributed throughout the colon (Table 3). No significant difference was found between the two groups in terms of the polyp morphological classification or location.

**Figure 2:**
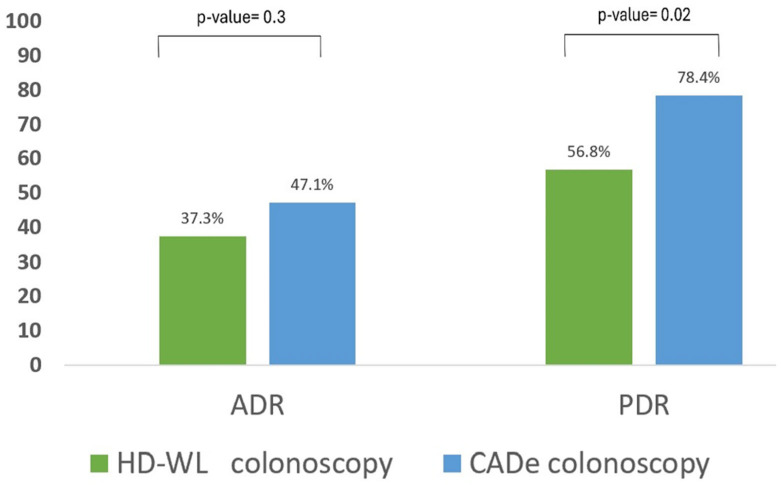
ADR and PDR (n = 102).

**Figure 3:**
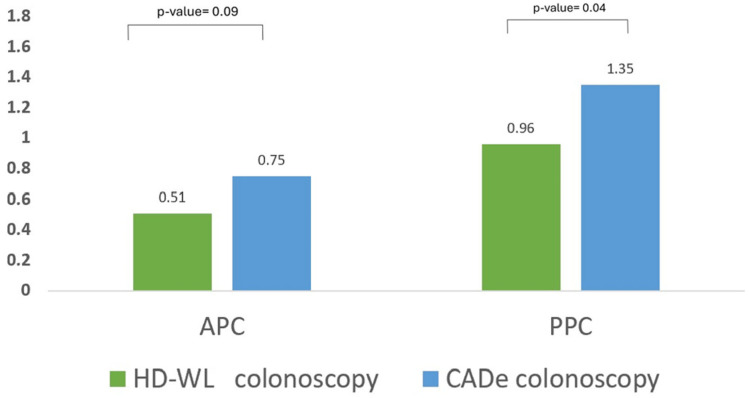
APC and PPC (n = 121).

***Polyp and adenoma detection*:** The use of CADe resulted in a non-significant increase in the ADR (primary outcome) compared to HD-WL colonoscopy alone (47.1% vs. 37.3%, *p* = 0.3) (Table 3). The use of CADe significantly increased the PDR compared to HD-WL colonoscopy (78.4% vs. 56.8%, *p* = 0.02) (Figure 2). Similarly, CADe was associated with a non-significant increase in the APC compared to HD-WL colonoscopy (0.75 vs. 0.51, *p* = 0.09), while it significantly increased the PDR (1.35 vs. 0.96, *p* = 0.04). (Figure 3).

***Polyp characterization:*** The use of an artificial intelligence system to characterize the polyp in vivo (CADx) was accurate in 55 polyps (79.7%), while the use of white light endoscopy and chromoendoscopy was able to accurately classify the polyp in 40 polyps (76.9%). This difference in characterization accuracy was not significant (*p* = 0.7).

## 4. Discussion

Colonoscopy is an effective method for detecting and removing precancerous colonic polyps that ultimately leads to a decreased incidence of CRC and its associated mortality. Even though colonoscopy is regarded as the “gold standard” method for the detection of early neoplastic colonic lesions, it can miss such lesions, which has been associated with an increased risk of interval CRC [2]. With the increasing incidence of CRC, especially among young adults [17], improved detection of early precancerous lesions is crucial to tackle such a public health problem. Despite the introduction of several technologies and devices to improve ADR among patients undergoing screening colonoscopy, the impact of such technologies remains unproven due to limited availability and the requirement to use extra devices that many physicians are not familiar with (e.g., distal caps) [18].

There has been a tremendous interest in incorporating technology and AI into gastroenterology to improve patient outcomes. Specifically, AI has been explored and implemented to aid in adenoma and polyp detection during colonoscopy. This technology has the potential to overcome some of the limitations of colonoscopy, mostly related to human factors such as endoscopist inattention or fatigue. Despite the theoretical advantage of AI in adenoma detection (i.e., CADe), the evidence of its benefit during screening and surveillance colonoscopy remains conflicting. Several RCTs, which were summarized in several meta-analyses, found higher ADR in the CADe group (by approximately 20%) [10,12]. However, these findings were not consistent in all studies, with several RCTs failing to find any improvement in ADR when applying AI during colonoscopy [8,9,19,20]. In one of the largest RCTs (n = 3213 patients) that assessed the impact of AI on ADR among Spanish patients presenting for a screening colonoscopy, it failed to show any improvement in ADR among the group of patients randomized to AI compared to a standard colonoscopy (aRR 1.06, 0.91–1.23) [9]. Similarly, our study found a non-significant increase in the ADR (RR = 1.26, 0.80–2.00) in the CADe group. Even though this 26% relative increase in ADR is similar to that reported by other RCTs, it failed to reach statistical significance. This could be related to the lack of power in our study (type 2 error), and a larger sample size may have identified a statistically significant difference, as discussed in more detail later. In our study, we identified a high ADR in the overall population (ADR 42.2%) despite limiting the inclusion to the average-risk population. The reason for this discrepancy between our study’s ADR and the previously reported ADR in the Kuwaiti population is unclear but could be related to a true improved ADR using newer colonoscopes, which have better optics compared to older colonoscopes. However, a true increase in adenomas in the Kuwaiti population over the last decade is another possibility that warrants further investigation. Irrespective of the reason for the discrepancy in the ADR between these studies, it is possible that using a higher baseline ADR for calculating sample size would have helped in detecting a true difference between the two arms, and future studies addressing this aspect in Kuwait should use our ADR as a new benchmark for the Kuwaiti population.

The APC, a measure of the number of adenomas detected per colonoscopy, was numerically, but not statistically, higher in the CADe group compared to the HD-WL colonoscopy group (0.75 vs. 0.51, *p* = 0.09). Similar to ADR, the lack of statistical significance may be related to the lack of power rather than a true lack of clinical significance. In fact, the study by Mangas-Sanjuan et al. discussed previously that failed to show a difference in ADR, the APC was significantly higher in the CADe group compared to HD-WL colonoscopy group (1.78 vs. 1.59, RR 1.12 (1.02–1.22)) [9]. Even though that study focused on a high-risk population (positive fecal immunohistochemistry test), it suggests that CADe can actually increase the number of adenomas detected in each colonoscopy.

When PDR was assessed, it became clear that CADe significantly increases the detection of colonic polyps (RR 1.38, 1.04–1.82). Similarly, the PPC was significantly higher in the CADe group compared to the HD-WL colonoscopy group, a finding that is consistent with previously published RCTs and meta-analyses [11,12]. However, the significance of this finding is of limited clinical importance as the majority of identified polyps are small, diminutive hyperplastic polyps that do not increase the risk of CRC, and the true effect of CADe on advanced adenomas remains unclear [9]. Nevertheless, it is clear that adopting AI into routine screening colonoscopy procedures did not affect the procedure time (insertion and withdrawal times), and it is an easy technology to adopt given its simplicity and wide availability with several AI systems already available in the market.

Besides detection of precancerous colonic polyps, one of the major challenges in clinical practice is polyp characterization in vivo. The decision on whether a detected polyp is a hyperplastic polyp, which has no malignant potential, or an adenoma that can transform into adenocarcinoma is an important one that has a significant impact on patient outcomes [21]. This distinction is particularly challenging among the small, diminutive polyps, where adenomatous changes might be more subtle. Therefore, in clinical practice, any detected polyp is routinely resected and sent for histopathological examination. Given that the vast majority of encountered polyps are diminutive (≤5 mm in size), such practice can place a tremendous burden on pathologists and increase the cost associated with screening colonoscopy. Therefore, a strategy of “resect-and-discard” has been suggested to reduce the associated costs without affecting patient outcomes, where an optical diagnosis is performed during colonoscopy and polyps that are deemed non-neoplastic can be resected without retrieval, or even not resected at all [22]. However, before such practices can be endorsed universally, a high-level of confidence in optical diagnosis is required. The most recent European Society of gastrointestinal endoscopy (ESGE) practice guidelines suggested limiting the “resect-and-discard” strategy of diminutive polyps to a situation where a technology can achieve a sensitivity of at least 90% in diagnosing adenomatous histology by optical assessment [23]. In the current study, the CAD system had an accuracy of 79.7% in characterizing polyp histology, which was not significantly different from that of HD-WL colonoscopy (accuracy 76.9%). The use of virtual chromoendoscopy, namely BLI in the current endoscopy system, did not seem to improve the polyp characterization significantly among patients randomized to the HD-WL colonoscopy, but this technology has an important role in assessing suspicious areas in large polyps [24,25]. The current AI system had good accuracy at classifying larger lesions (>10 mm), but these represented a small proportion of polyps that are detected in clinical practice, all of which require resection irrespective of the predicted histology. However, when it comes to the more common diminutive polyps, AI systems still struggle to correctly classify some of the adenomatous polyps where they are still labeled as “hyperplastic”. Therefore, improvement in AI technology is required to increase the accuracy of polyp characterization before it can be recommended routinely for the implementation of a “resect-and-discard” strategy.

Our study has several strengths that warrant highlighting. First, it is the first RCT in the region to study and assess the impact of AI on screening colonoscopy in local populations, providing important information that can be used for future studies in the region. The use of a standardized protocol and AI system allowed us to obtain a homogenous result with no patients lost to follow-up and without deviation from the study protocol. All detected polyps were resected and sent for histopathological examination by expert pathologists providing pathological confirmation of polyp histology. We ensured that only patients who were asymptomatic and had an average risk for CRC were included in this study to increase the applicability of the study findings to screening programs. One of the potential limitations of our study is the limited power given the number of patients included in the study (102 patients). The sample size calculation was based on the reported ADR in Kuwait, namely 10%, as was previously reported by Alenezi et al. [15]. Based on the available ADR and the assumptions we made as discussed in the methods section, the required sample size was 86 in total to find an absolute difference of 25% in ADR. However, in the current study, it became clear that the previously reported ADR of 10% is significantly lower than the current ADR in Kuwait, with the overall ADR in the current study being 42.2%. This high ADR is likely related to the performance of all colonoscopies by a single experienced endoscopist without the involvement of any trainees; however, other factors such as a true increase in the prevalence of adenomas in the general Kuwaiti population over the last decade is another important consideration. This high adenoma detection rate likely resulted in an underpowered study to detect a true difference in ADR between the two arms (type 2 error). Nevertheless, this underscores the importance to our study, which provided a new and updated ADR for the Kuwaiti population that should be considered as the new benchmark when it comes to future studies and auditing of the CRC screening program in Kuwait. Furthermore, the impact of AI on ADR among endoscopists with low ADR is unclear but could be more significant than among endoscopists with high baseline ADR. Another limitation of this study is the single-center design, but given the homogenous Kuwaiti population, this likely had little impact on the generalizability of our findings. We did not assess the cost-effectiveness of implementing this technology in screening programs routinely, but such cost-effectiveness analysis should only be implemented once the efficacy of this technology is proven. We used a single AI system in this study (CAD EYE, Fujifilm), and the findings of our study cannot be applied to other AI systems.

In conclusion, CADe resulted in a non-statistically significant increase in the ADR among the average-risk Kuwaiti population undergoing screening colonoscopy, but significantly increased the detection of colonic polyps overall, mostly diminutive hyperplastic polyps. AI systems still lack adequate polyp characterization accuracy to be recommended in lieu of formal histopathological examination. Further multicenter studies are required to detect a true effect of AI on ADR in the Kuwaiti population using the new baseline ADR described in our study.

## Figures and Tables

**Table 1:** Baseline characteristics of included patients and procedures (n = 102).

	Overall (n = 102)	HD-WL Colonoscopy (n = 51)	CADe-Assisted Colonoscopy (n = 51)	*p*-Value
**Male, n (%)**	51 (50)	21 (41.2)	30 (58.8)	0.08
**Age, mean (SD), years**	52.8 (8.2)	54.5 (8.3)	51.1 (7.7)	0.03
**Weight (KG), mean (SD)**	79.5 (14.2)	79.9 (16.3)	79.0 (11.9)	0.73
**Indication, n (%)**				1.00
- Screening	96 (94.1)	48 (94.1)	48 (94.1)	
- Surveillance	6 (5.9)	3 (5.9)	3 (5.9)	
**Use of antithrombotics, n (%)**	5 (4.9)	3 (5.9)	2 (3.9)	0.65
**BBPS colon cleansing scale, median (IQR)**	8 (6–9)	8 (6–9)	8 (6–9)	1.00
**Insertion time, mean (SD), s**	341.2 (186.5)	359.9 (136.8)	322.5 (225.4)	0.31
**Withdrawal time, mean (SD), s**	525.9 (115.0)	509.4 (104.2)	542.4 (123.8)	0.15
**IC valve intubation, n (%)**	94 (92.2)	44 (86.3)	50 (98.0)	0.03
**Adverse Events, n (%)**	0 (0)	0 (0)	0 (0)	1.00

**Table 2:** Polyp characteristics (n = 121).

	Overall (n = 121)	HD-WL Colonoscopy (n = 52)	CADe-Assisted Colonoscopy (n = 69)	*p*-Value
**Polyp size (mm), mean (SD)**	4.18 (5.1)	4.19 (6.3)	4.17 (4.0)	0.98
**Polyp histology, n (%)**				0.81
Hyperplastic	56 (46.3)	23 (44.2)	33 (47.8)	
TA with LGD	57 (47.1)	25 (48.1)	32 (46.4)	
TA with HGD	0 (0)	0 (0)	0 (0)	
TVA with LGD	3 (2.5)	1 (1.9)	2 (2.9)	
TVA with HGD	1 (0.8)	1 (1.9)	0 (0)	
Inflammatory	4 (3.3)	2 (3.9)	2 (2.9)	
Malignant	0 (0)	0 (0)	0 (0)	
**Paris classification, n (%)**				0.39
Ip	1 (0.8)	0 (0)	1 (1.45)	
Is	15 (12.4)	6 (11.5)	9 (13.0)	
Ips	1 (0.8)	0 (0)	1 (1.45)	
IIa	101 (83.5)	46 (88.5)	55 (79.7)	
IIb	3 (2.5)	0 (0)	3 (4.4)	
IIc	0 (0)	0 (0)	0 (0)	
III	0 (0)	0 (0)	0 (0)	
**Polyp location, n (%)**				0.27
Rectum	15 (12.4)	6 (11.5)	9 (13.0)	
Sigmoid	20 (16.5)	8 (15.4)	12 (17.4)	
Left colon	26 (21.5)	10 (19.2)	16 (23.2)	
Transverse colon	32 (26.5)	18 (34.6)	14 (20.3)	
Right colon	23 (19.0)	10 (19.2)	13 (18.8)	
cecum	5 (4.1)	0 (0)	5 (7.2)	

**Table 3:** Primary and secondary outcomes (n = 102).

	Overall (n = 102)	HD-WL Colonoscopy (n = 51)	CADe-Assisted Colonoscopy (n = 51)	*p*-Value	Risk Ratio (95%CI)
**Adenoma detection rate (ADR), n (%)**	43 (42.2)	19 (37.3)	24 (47.1)	0.32	1.26 (0.80–2.00)
**Polyp detection rate (PDR), n (%)**	69 (67.6)	29 (56.8)	40 (78.4)	0.02	1.38 (1.04–1.82)
**Adenoma per colonoscopy, mean (SD)**	0.63 (0.89)	0.51 (0.78)	0.75 (0.99)	0.09	-
**Polyp per colonoscopy, mean (SD)**	1.15 (1.15)	0.96 (1.20)	1.35 (1.07)	0.04	-
Accuracy in characterization, n (%) *	95 (78.5)	40 (76.9)	55 (79.7)	0.71	-

* Per polyp assessment (n = 121).

## Data Availability

All data that support the findings of this study are available from the corresponding author upon reasonable request.

## Abbreviations

ADR	Adenoma detection rate
AI	Artificial intelligence
APC	Adenoma per colonoscopy
CADe	Computer-aided detection
CRC	Colorectal cancer
HD	High definition
PDR	Polyp detection rate
PPC	Polyp per colonoscopy
WL	White light
